# The Abrolhos Nominally Herbivorous Coral Reef Fish Acanthurus chirurgus, Kyphosus sp., Scarus trispinosus, and Sparisoma axillare Have Similarities in Feeding But Species-Specific Microbiomes

**DOI:** 10.1007/s00248-024-02423-x

**Published:** 2024-08-31

**Authors:** Cristiane Thompson, Raphael Silva, Fernando Z. Gibran, Leonardo Bacha, Mayanne A. M. de Freitas, Mateus Thompson, Felipe Landuci, Diogo Tschoeke, Xiao-Hua Zhang, Xiaolei Wang, Wenbin Zhao, Pedro Vianna Gatts, Marcelo Gomes de Almeida, Carlos Eduardo de Rezende, Fabiano Thompson

**Affiliations:** 1 Laboratory of Microbiology, Instituto de Biologia, Universidade Federal do Rio de Janeiro (UFRJ), Av. Carlos Chagas Filho 373, Sala 102, Bloco A, CCS/IB/BIOMAR, Lab. de Microbiologia, Cidade Universitária, Rio de Janeiro, RJ CEP 21941-599 Brazil; 2 Centro de Ciências Naturais e Humanas (CCNH), Universidade Federal do ABC (UFABC), São Bernardo Do Campo, São Paulo, Brazil; 3 Biomedical Engineer Program, COPPE, Universidade Federal do Rio de Janeiro (UFRJ), Rio de Janeiro, Brazil; 4 Microbial Oceanography Lab, Ocean University of China, Qingdao, China; 5 Laboratory of Environmental Sciences (LCA), Center of Biosciences and Biotechnology (CBB), Universidade Estadual do Norte Fluminense Darcy Ribeiro (UENF), Campos Dos Goytacazes, Brazil

**Keywords:** Fish, Coral reefs, Gut microbiome, Isotopes

## Abstract

Coral reefs rely heavily on reef fish for their health, yet overfishing has resulted in their decline, leading to an increase in fast-growing algae and changes in reef ecosystems, a phenomenon described as the phase-shift. A clearer understanding of the intricate interplay between herbivorous, their food, and their gut microbiomes could enhance reef health. This study examines the gut microbiome and isotopic markers (δ^13^C and δ^15^N) of four key nominally herbivorous reef fish species (*Acanthurus chirurgus*, *Kyphosus* sp., *Scarus trispinosus*, and *Sparisoma axillare*) in the Southwestern Atlantic’s Abrolhos Reef systems. Approximately 16.8 million 16S rRNA sequences were produced for the four fish species, with an average of 317,047 ± 57,007 per species. Bacteria such as Proteobacteria, Firmicutes, and Cyanobacteria were prevalent in their microbiomes. These fish show unique microbiomes that result from co-diversification, diet, and restricted movement. Coral-associated bacteria (*Endozoicomonas*, *Rhizobia*, and *Ruegeria*) were found in abundance in the gut contents of the parrotfish species *Sc. trispinosus* and *Sp. axillare*. These parrotfishes could aid coral health by disseminating such beneficial bacteria across the reef. Meanwhile, *Kyphosus* sp. predominantly had Pirellulaceae and Rhodobacteraceae. Four fish species had a diet composed of turf components (filamentous Cyanobacteria) and brown algae (*Dictyopteris*). They also had similar isotopic niches, suggesting they shared food sources. A significant difference was observed between the isotopic signature of fish muscular gut tissue and gut contents, pointing to the role that host genetics and gut microbes play in differentiating fish tissues.

**Supplementary Information:**

The online version contains supplementary material available at 10.1007/s00248-024-02423-x.

## Introduction

Herbivorous fish play a crucial role in maintaining the health of coral reefs [5, 30, 77]. Known as the primary regulators of algae cover, these fish can eradicate over 90% of daily algae and turf production in shallow coral reefs [8]. Overfishing, however, has led to a decline in herbivores, promoting the growth of fast-spreading organisms like algae that dominate the sea floor, a phenomenon referred to as the phase-shift process [47]. Anthropogenic stress, i.e., human-induced nutrient pollution, threatens coral reefs by causing endogenous microbial shifts in wild reef fish [19]. Coral reef herbivory is complex, involving an array of primary consumers whose feeding habits, diets, morphology, physiology, and ecological functions greatly vary [17, 48]. Evolutionary developments like gizzard-like stomachs and bristle-like teeth have enabled herbivorous fish to feed on specific producers of coral reefs [63, 77]. Diet strongly influences the gut microbiome of surgeonfishes (family: Acanthuridae) [55]. For instance, while some *Acanthurus* fish, as *A. chirurgus*, feed mainly on detritus, macroalgae, turf, and invertebrates, and are considered sediment suckers [28, 77], some parrotfish, as *Scarus trispinosus* and *Sparisoma axillare*, consume corals, crustose coralline algae (CCAs), turf, epilithic, and endolithic Cyanobacteria, and microalgae [14, 32, 34]. Macroalgae is not their primary food source but is consumed incidentally in parrotfishes [14]. Both parrotfish and *Acanthurus* species significantly influence the dynamics of detritus and sediment [77]. Kyphosus feed mainly on macroalgae [9] with gut and mouth structures well adapted for this diet. Although they also consume invertebrates such as calanoid copepods, likely opportunistically, their feeding behavior varies with the age and size of the fish [72].

Parrotfish and some grazing acanthurids as *A. chirurgus* use a mechanical grinding process in their pharyngeal mill or gizzard-like stomach, respectively, relying mainly on swift gut throughput and protein, lipid, and soluble carbohydrate digestion [14]. In contrast, kyphosids have a longer gut that may rely on refractory carbohydrate fermentation [56]. *Acanthurus* species have acidic stomachs, ideal for digesting tough algal material. Algae-derived carbohydrate fermentation is a key energy source, producing short-chain fatty acid (SCFA) [45]. SCFA production can also occur in the final part of the intestine (hindgut chamber or caecal pouch) in *Kyphosus vaigienses* [67]. Past studies in Lizard Island, Australia, show that *K. vaigiensis* predominantly consumes phaeophytes, while *K. cinerascens* eats a substantial amount of rhodophytes [13].

The bacteroidota genera *Alistipes* and *Rikenella* are found abundantly in the furthest part of the lumen section, where SCFA levels are at their peak. These bacteria play a crucial role in breaking down seaweed into compounds beneficial for the fish [76]. In addition, metagenomic analyses of *K. sydneyanus* hindgut contents have revealed the degradation pathways for vital algae dietary substrates like mannitol, alginate, laminarin, fucoidan and galactan, agar, and carrageenan [76]. Moreover, a noticeable uptake of fermentation products such as acetate in *K. sydneyanus* hindgut was observed [56]. These variations in fermentation by-products may explain the isotopic differentiation seen in fish tissue. The gut of fish is known to house many microbial cells (1.71 × 109 g^−1^ dry wt feces; [73]). A recent study of the *Acanthurus* gut microbiome discovered the presence of Lachnospiraceae, a microbe thought to facilitate the digestion and absorption of carbohydrate-dense brown macroalgae [71]. The gut microbiome of seagrass-specializing parrotfish (*Calotomus spinidens*) in Fiji’s reef areas and surgeonfish (*Acanthurus nigricauda*, *Ctenochaetus striatus*) in the Florida Keys was predominantly Proteobacteria [52]. In contrast, the gut of *Paracirrhites bicolor*, *P. arcatus*, *P. xanthus*, and *P. nisus* fish observed in the Line Islands of the Republic of Kiribati was teeming with *Firmicutes* [37]. It is important to note that the *Paracirrhites* species consists of piscivores/invertivores [84], which have very different diets compared to the herbivorous studied here. Notably, *Acanthurus triostegus* from Australia’s Great Barrier Reef had a high quantity of *Firmicutes*, Epulopiscium [60]. This microbe also held dominance in the gut of seven herbivorous coral reef fish varieties in the South China Sea [38]. Regrettably, data detailing the microbiome composition, fermentation triggers, and possible symbionts of herbivores inhabiting Southwestern Atlantic reefs remains scarce.

Fish contribute to reef fertilization through a steady supply of microbe-rich feces [40]. An example is the parrotfish cyanobacteria diet that enriches the coral ecosystem. Besides that, parrotfishes have the potential to disperse Symbiodinium, the photosynthetic microalgae indispensable for stony coral survival [10, 42, 58]. Corallivorous fish, such as parrotfish, may affect the health of coral by sharing microbial symbionts by biting coral and distributing their waste across reefs [66]. Thus, parrotfish corallivory could be a beneficial process for disseminating helpful bacteria over reefs. This activity has been linked to the transmission of mutualistic bacteria [41]. Nevertheless, parrotfish may also contribute to reef bioerosion and sediment production and movement [51, 62]. Herbivorous fish, including *Scarus* and *Acanthurus*, consume cyanobacterial turf and feces of planktivorous fish [65]. Coprophagy, or feces-eating, apparently provides significant nutrient and energy intake [65]. Additionally, climate change could trigger the growth of potentially disease-causing bacteria in reef fish [25]. However, the composition of the feces of Southwestern Atlantic coral reef fish remains largely unknown.

Metagenomics (eDNA) analysis of gut contents infers that herbivorous reef fish may have a varied diet and diversified feeding behavior [57]. In one recent study conducted in an upwelling region, *Acanthurus chirurgus* and *Sparisoma axillare* shared some similarities in diets comprised of red calcareous articulated algae, red corticated algae, detritus, and diatoms [9]. These fish also consumed other common food items like Cyanobacteria, red and green filamentous algae [9]. The δ^15^N signatures of their food sources, potentially including macroalgae, suspended particulate matter, and sediment (ranges from 6 to 7‰), were consistent with those found earlier in macroalgae (6.1‰), suspended particulate matter (6.2 − 6.5‰), and sediment organic matter (6.4 − 7.1‰). However, despite having similarities in diets and gut contents, *A. chirurgus* (δ^13^C: − 18.7 ± 0.3‰; δ^15^N: 12.3 ± 0.2‰) and *Sp. axillare* (δ^13^C: − 16.0 ± 0.1‰; δ^15^N: 10.8 ± 0.2‰) displayed different isotopic signatures, possibly due to differences in their genetics and/or microbiomes, such as through microbial fermentation [9]. Different microbiomes may have different fermentation pathways and therefore different fermentation products. The four species belong to different genera and have distinct intestinal features, leading to unique feeding biologies. Nevertheless, they all share the common trait of being nominally herbivorous. These two herbivorous fish species (*A. chirurgus* and *Sp. axillare*) feed on many types of coral reef substrates, suggesting similar microbial compositions. However, additional research on fish microbiomes is required to validate this hypothesis.

In the Abrolhos Reef systems of the Southwestern Atlantic Ocean (Brazil), there are four nominally herbivorous fish: *Scarus trispinosus*, *Sp. axillare*, *Acanthurus chirurgus*, and *Kyphosus* sp. [31–33, 35]. Among these, the greenbeak parrotfish (*Sc. trispinosus*) is the largest, while the gray parrotfish (*Sp. axillare*) is the smallest. Individuals of *Sc. trispinosus* larger than 30 cm are excavators [51], biting corals, turf, and CCAs, probably searching for epilithic and endolithic Cyanobacteria, and microalgae (see [14]), while individuals smaller than 30 cm are scrapers of the same type of substrates [28, 32, 34, 51]. In contrast, individuals of *Sp. axillare* are browsers, biting mainly turf and macroalgae [9, 28, 32, 34].

Over the past decade, the abundance of these four reef fish species has declined in Abrolhos, coinciding with noticeable phase shifts [7, 31]. Following the sharp decline of large carnivorous reef fishes, parrotfishes were progressively targeted by commercial fisheries in Brazil, resulting in a global population decline of 50% for *S. trispinosus* [36]. A significant development related to this is the increase in turf algae, which covers over 60% of the benthic cover in some areas of the Abrolhos Bank [7, 81]. Along with the coral reef phase shift, there has been a significant increase in the proportion of fermentative bacteria (*Rikenella*, *Akkermansia*, *Desulfovibrio*, *Brachyspira*) in the gut of reef fishes particularly in the Siganidae family present in the Indian Ocean [12]. Consequently, the changes in the abundance of herbivorous fish also impact the coral reef benthic communities [61, 68]. However, more information is needed regarding the composition of the microbiome and potential symbionts of the herbivorous fish in Abrolhos.

This study sought to investigate the microbiome composition of the four nominally herbivorous reef fish of the Abrolhos Bank. We examined microbial diversity (using 16S rRNA Illumina sequencing) from various sections of the fish’s gut. We also analyzed the isotopic composition of carbon (δ^13^C) and nitrogen (δ^15^N) in the fish tissue and gut content, which helped to identify potential food sources. The gut anatomy of *Sc. trispinosus* and *Sp. axillare* is homogeneous, naturally with anatomy composed of three major sections (stomach absent). On the other hand, *Khyphosus* sp. and *A. chirurgus* have five distinct sections in their gut. However, one aspect that remains unclear is how the microbiome could vary throughout their gut.

## Material and Methods

### Fish Gut Microbiome Analysis in the Abrolhos Coral Reefs: Collection Method and Initial Findings

**Fig. 1:**
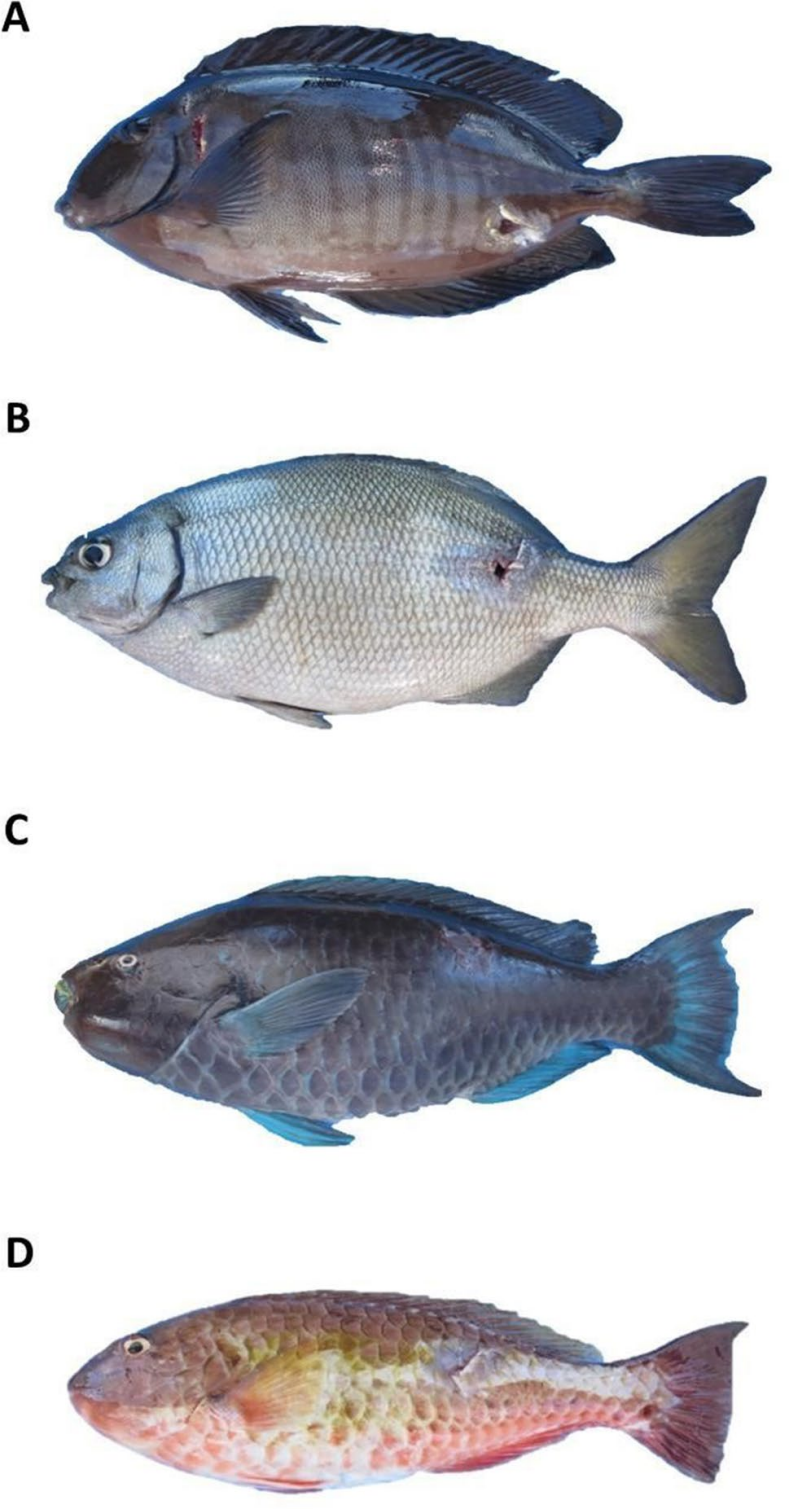
Common Abrolhos herbivorous reef fish. **A** *Acanthurus chirurgus*; **B** *Kyphosus* sp.; **C** *Scarus trispinosus*; **D** *Sparisoma axillare*

**Fig. 2:**
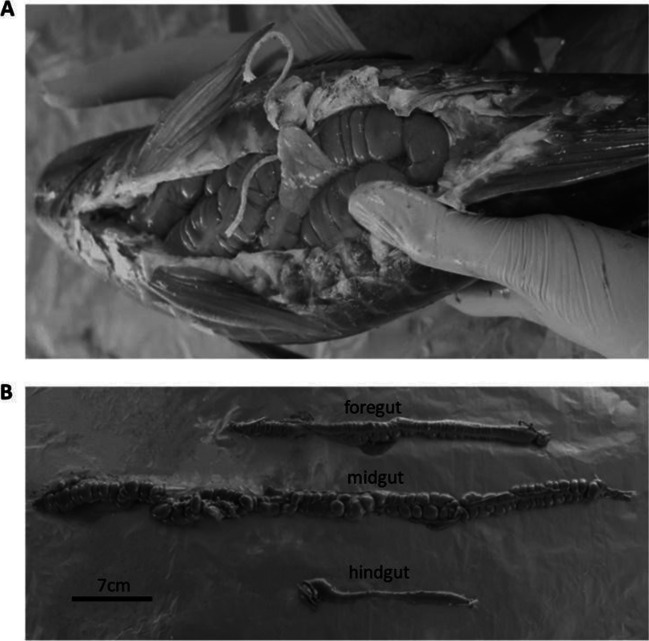
Fish gut. **A** A fish individual of *Scarus trispinosus* with a ventral cut, showing the digestive tract inside the body. **B** Foregut, midgut, and hindgut of *S. trispinosus*. The parrotfishes *Sc. trispinosus* and *Sparisoma axillare* lack a stomach

The Abrolhos region, which extends from southern Bahia to northern Espírito Santo, houses the largest and most diverse coral reefs in the South Atlantic [31]. For our study, fish were collected on February 10th, 2016 (*n* = 8), and October 15th, 2017 (*n* = 8), at a depth of approximately 10 m using a speargun and immediately euthanized by pithing. We ensured the gathering of two specimens per species each season, yielding four specimens per species, with lengths (from the tip of the snout to the posterior end of the last vertebra) varying from 22 to 49 cm. All the fish specimens (*n* = 16) were extracted from Abrolhos Archipelago (Fig. 1). This study received authorization from Parque Nacional Marinho dos Abrolhos/ICMBio (Brazilian Environmental Agency) – scientific research and collecting permits SISBIO 50872–1, 50,872–2. The digestive tracts of these fish followed the division as previous reports describe [56] and their natural anatomy. *Sc. trispinosus* and *Sp. axillare* guts have three major portions, foregut (pH = 6.5–8), midgut (pH = 7–8), and hindgut (pH = 7–8) (Fig. 2). On the other hand, *Kyphosus* sp. and *A. chirurgus* have five portions each. For *Kyphosus* sp., a foregut/stomach (pH = 3.5–8), anterior intestine (pH = 7.5–8.5), midgut (pH = 6.5–9), end midgut (pH = 7–8), and hindgut (pH = 7–7.5). For *A. chirurgus*, a foregut*/*anterior stomach (pH = 7–8), a gizzard-like stomach (pH = 7.5–8.5), anterior intestine (pH = 5.5–7), midgut (pH = 6–7.5), and hindgut (pH = 6.5–8) (Fig. 2). Following collection, the digestive tract of each fish specimen was cut with sterilized material and samples of gut muscle tissue as well as gut contents from each aforementioned gut portion were separated. We also measured pH for each gut portion. Samples were secured in 2 mL cryovials and immediately preserved in liquid nitrogen on board for subsequent laboratory analysis. We estimated the colony-forming units (CFUs) in the gut contents on marine agar (Difco2216), and counts were determined after a 48-h incubation period at 24 °C for all specimens. Divers collected some of the most abundant types of coral species and algae substrates in the region, which included *Montastraea cavernosa*, *Mussismilia braziliensis*, turf, and rhodoliths, without no harm to the ecosystem. These substrates (food sources) were also taken to liquid nitrogen inside falcon tubes, for subsequent isotope analysis.

### Fish DNA Extraction and 16S rRNA Illumina Sequencing

The DNA extraction from fish gut contents, weighing 1 g, was undertaken as per the method specified by Garcia et al. [39] using the Power Soil Kit (Qiagen) according to the manufacturer’s specifications. The 16S rRNA gene amplification was achieved through a two-step PCR approach using the V3-V4 region primers 16S_341F (5′-TCGTCGGCAGCGTCAGATGTGTATAAGAGACAGCCTACGGGNGGCWGCAG-3′) and 16S_805R (5′-GTCTCGTGGGCTCGGAGATGTGTATAAGAGACAGGACTACHVGGGTATCATCC-3′). This amplification program involved an initial 5-min denaturation at 95 °C, followed by 34 cycles of 30 s at 95 °C, 45 s at 55 °C, and 90 s at 72 °C. It concluded with a final 10-min extension at 72 °C. Once prepared, Illumina barcodes were added to each sample using Nextera barcoding primers, and then sequenced as per the Illumina protocol for 16S rRNA, specifically the 16S Metagenomic Sequencing Library Preparation. The Illumina MiSeq platform was used for sequencing. These data have been deposited in GenBank, https://ncbi.nlm.nih.gov, under BioProject accession number PRJNA1131363: Biosample SAMN42278458 to SAMN42278510.

### Bioinformatic Analysis

The sequence quality was assessed using Multiqc [22], with a cutoff point at 150 bp, and the trimmomatic was employed to eliminate the bases of low-quality sequences. The software Qiime2 Version 2020.2 [4] was utilized to analyze the sequences. Raw data were imported and demultiplexed using qiime2 tools import plugin. In order to improve data quality and ensure that only relevant biological sequences are retained, primers were removed using the qiime cutadapt trim-paired plugin with an error-rate = 0 and then forward and reverse sequences were joined (qiime vsearch join-pairs). The joined sequences were filtered using the qiime quality-filter plugin to retain only those with a Q-score ≥ 20. Before taxonomic classification, sequences were dereplicated (using qiime vsearch dereplicate-sequences) and clustered with a minimum identity similarity of 97% (a threshold less sensitive to sequencing errors). After this step, chimeras were removed, and taxonomic classification was performed using the Silva 132 database (V. Silva-132–97-nb).

**Fig. 3:**
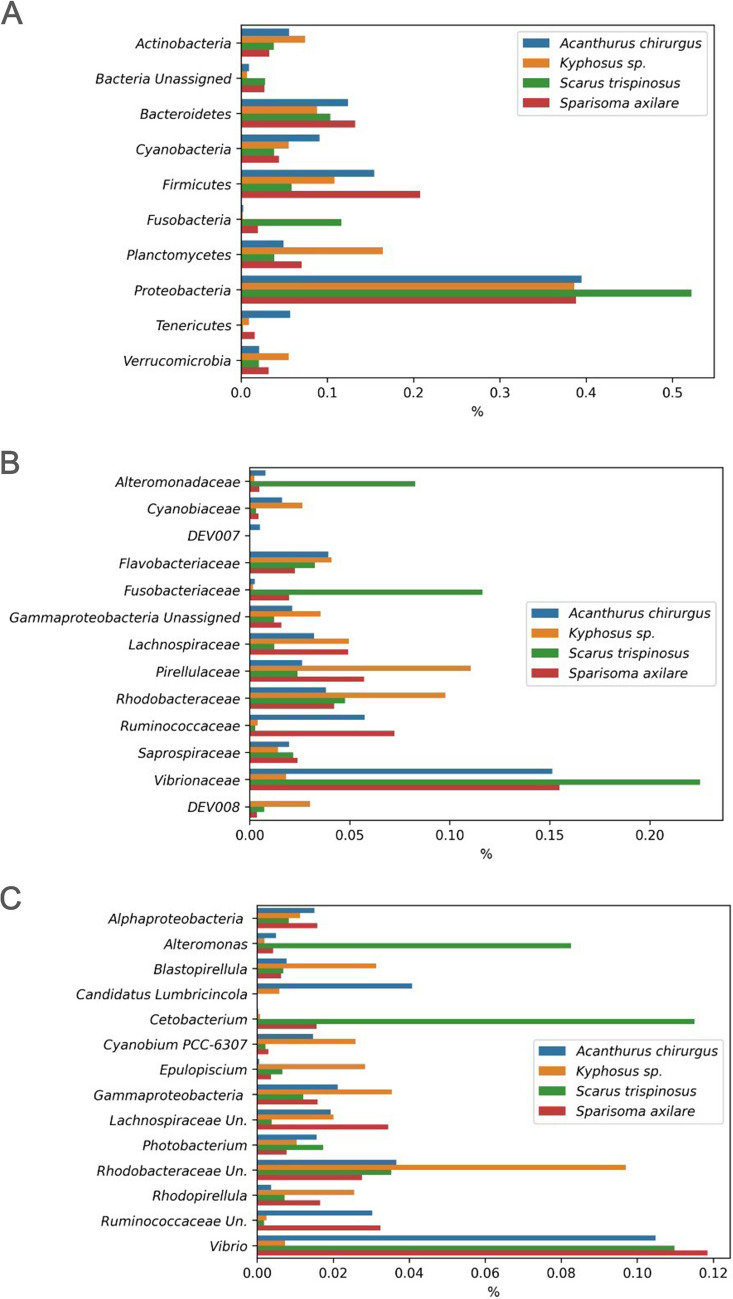
Relative abundance of microbial phyla (**A**), family (**B**), and genera (**C**). Proteobacteria and Firmicutes appear as the most representative phyla. Higher ranks listed in the genus refer to unassigned therein. *Vibrio* and unassigned genera in Rhodobacteraceae were abundant groups. *Scarus trispinosus* had high abundance of *Alteromonas*. Values are mean ± std error. Unassigned genera (Rhodobacteraceae: *p*-value = 0.003; Gammaproteobacteria: *p*-value = 0.01), *Blastopirellula* (*p*-value = 0.001), *Rhodopirellula* (*p*-value = 0.004), Vibrionaceae genera (*p*-value = 0.002) were significantly different among fish species

For statistical analyses and graphical visualizations (microbial investigation and isotope analysis in Figs. 3 up to 8), the 4 specimens of each fish species were considered. The approach focused on the most abundant total microbial composition in the overall digestive system or, when necessary, in the specific portions of the intestinal tract. All analyses were performed using the R software. Finally, to explore the evolutionary driving forces of fish microbiomes, we applied the null model [75] calculated via the picante, ecodist, and iCAMP packages in R (v4.3.2). Recently, neutral theory primarily focuses on species-rich communities (e.g., coral reefs and tropical forests) with some rare species having a greater individual stochasticity [11], stochastic processes have also been demonstrated as important roles in the community structure, including immigration, birth, death, and diversification [26, 43, 85].

### Stable Isotope Analysis (δ13C and δ.15N)

Fish gut content’s δ^13^C and δ^15^N were evaluated as outlined in prior work [54]. These samples were preserved in sterile liquid nitrogen falcon tubes and subsequently brought to the lab, where they were kept frozen (− 20 °C) before assessment. These lyophilized samples went through the process of grinding in a crucible until they became a fine powder. Approximately 0.5 to 1 mg of gut content, gut muscular tissue, and potential fish food sources (*Mo. cavernosa*, *Mu. braziliensis*, turf, and rhodoliths) were used. For gut content and food sources, acidification was performed by adding HCl (2 M) to remove inorganic carbon (CaCO3) [6, 46]. A Flash 2000 Elemental Analyzer with a CONFLO IV interface connected to a Delta V Advantage isotope ratio mass spectrometer (Thermo Scientific, Germany) was used to study all samples’ elemental and isotopic composition. Analytical blanks and urea analytical standards were utilized during analysis, with known isotopic compositions. Certified isotopic standards were used to control the accuracy of gut content samples in every set of ten samples. The carbon and nitrogen content were calculated as a percentage, with detection thresholds of 0.05% and 0.02%. Carbon and nitrogen isotope ratios were given in δ notation, expressed as ‰ in response to Pee Dee Belemnite (PDB) and atmospheric nitrogen, and were determined utilizing a specific equation. The study’s accuracy was checked every ten samples. A carbon-to-nitrogen atomic ratio of ≥ 3.5 indicated a lipid bias in the stable isotope analysis, especially regarding carbon isotopes [64]. Hence, to maintain reliable interpretation, mathematical corrections developed by Logan et al. [53] were applied to δ^13^C calculations (δ^13^C = 0⋅967 × δ^13^C_bulk_ + 0⋅861).

The mean and standard deviations for δ^13^C and δ^15^N were computed for both gut and fish tissue from some potential food source (*Mo. cavernosa*, *Mu. braziliensis*, turf, and rhodoliths). The normality and homoscedasticity of the data set were verified using the Shapiro–Wilk and Levene’s tests, respectively. Variations were examined using Type III ANOVA, which is suitable for unbalanced data [70]. Any differences noted were significant if the *p-*value was below 5%. For each herbivorous fish species, discrimination factors were calculated between the gut content and fish tissue based on their functions—digestion and assimilation, respectively. This was accomplished using the equation: *Ɛ* = *δ*substrate − *δ*product / (*δ*product + 1). Here, *Ɛ* represents the enrichment factor, *δ*substrate indicates δ^13^C and δ^15^N of gut contents, and *δ*product denotes δ^13^C and δ^15^N of tissue contents. The isotopic niche breadth for each species was calculated using the Stable Isotope Bayesian Ellipses tool in R (SIBER). This utilized the standard ellipse area corrected for small sample sizes (SEAc; *n* ≤ 30) [44].

The isotopic niche metrics, which detail the properties of the isotopic space filled by each fish species, were estimated. These include (1) maximum δ^15^N range (NR), a larger range indicates more trophic levels and a higher degree of trophic diversity; (2) maximum δ^13^C range (CR), an increased range suggests a higher diversity of basal sources; (3) total area of isotopic niche (TA), this represents the total niche space occupied; (4) corrected standard ellipses area for small sample size (SEAc), this reflects the isotopic niche width; (5) mean Euclidean distance from the centroid (CD), greater distances suggest a high average degree of trophic diversity within the food web; (6) mean Euclidean distance to the nearest neighbor (MNND), small values denote similar trophic ecology; and (7) standard deviation of NND (SDNND), low values suggest a more evenly distributed trophic niche [49, 50].

## Results and Discussion

### Microbiome Diversity and Temporal Dynamics in Herbivorous Fish Species from the Abrolhos Coral Reefs

**Fig. 4:**
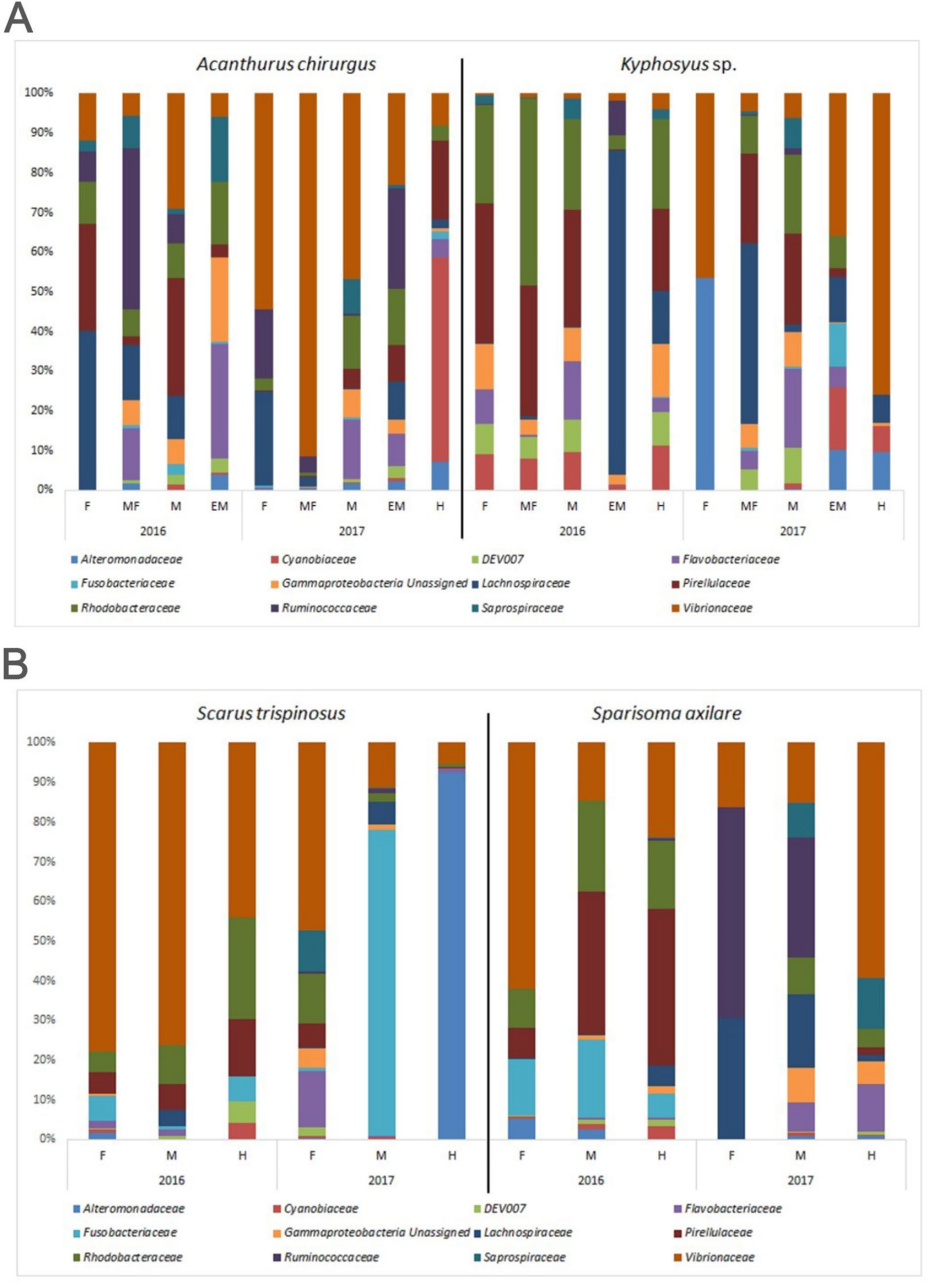
Major microbial families in *Acanthurus chirurgus* and *Kyphosus* sp. (**A**), with five divisions of the intestinal tract (**A**), and bellow, *Sparisoma axillare* with *Scarus trispinosus* (**B**), having three natural divisions of tract (**B**), in 2016 and in 2017

We generated a total of 16.80 million 16S rRNA sequences across four fish species, resulting in an average of 317,047 ± 57,007 sequences per library (*n* = 53 libraries) (Supplementary Table 1). Proteobacteria, *Firmicutes*, and Cyanobacteria made up about 90% of the microbiomes of *A. chirurgus* and *Sp. axillare* (Fig. 3A). However, each fish species displayed a unique microbiome. Specifically, *A. chirurgus* and *Sp. axillare* demonstrated higher quantities of Ruminococcaceae. On the other hand, *Sc. trispinosus* exhibited an increased abundance of Alteromonadaceae and Fusobacteriaceae, while *Kyphosus* sp. showed greater amounts of Pirellulaceae and Rhodobacteraceae (Fig. 3B). Apart from this, we also observed temporal changes in the gut microbe compositions of the fish taxa between february 2016 and october 2017 (Fig. 4). These annual variations in microbiome profiles within the same species may be attributed to changes in diet due to seasonal food availability. For example, *Kyphosus vaigiensis* generally consumes *Sargassum* in the summer and switches to *Dictyota*, *Plocamium*, and *Gelidium* during winter [9].

### Microbiomes Reveal a Shared Pool of Food Items Among Herbivorous Fish

All four studied fish species exhibited identifiable sequences of *Dictyopteris undulata* in their digestive systems, although *A. chirurgus* presented the highest amount. The finding of this brown alga’s sequences in the fishes’ systems implies active herbivory. Also, Cyanobacteria ranging between 1 and 2% were mainly composed of *Cyanobium* PCC-6307 in *Kyphosus* and *Acanthurus* species (Fig. 3C). Other varieties such as *Trichodesmium* IMS101, *Schizothrix* LEGE 07164, *Cylindrospermopsis* CRJ1, *Synechococcus* PCC-7336, *Phormidium* MBIC10003, *Synechococcus* CC9902, *Cyanothece* sp. WH 8902, and *Arthrospira* PCC-7345 were also detected. These Cyanobacteria might be elements of turfs [81]. The higher presence of Cyanobacteria in *A. chirurgus* and *Kyphosus* sp. could signal pronounced herbivory over turf. Concurrently, families like Ruminococcaceae were more prevalent in *A. chirurgus* and *Sp. axillare*, while Vibrionaceae, Alteromonadaceae, and *Fusobacterium* were more present in *Sc. trispinosus*. CFUs measured on marine agar revealed greater counts in *A. chirurgus* and *Kyphosus* sp. compared to *Sc. trispinosus* and *Sp. axillare*. This could underpin that *A. chirurgus* and *Kyphosus* sp. might depend on microbial fermentation. Both *Khyphosus* sp. and *A. chirurgus* exhibit five distinct gut portions, while *A. chirurgus has also a* gizzard-like stomach. This higher anatomical complexity offers further backing for the fermentation hypothesis [17].

### Contribution of the Gut Microbiome to Fish Health

**Fig. 5:**
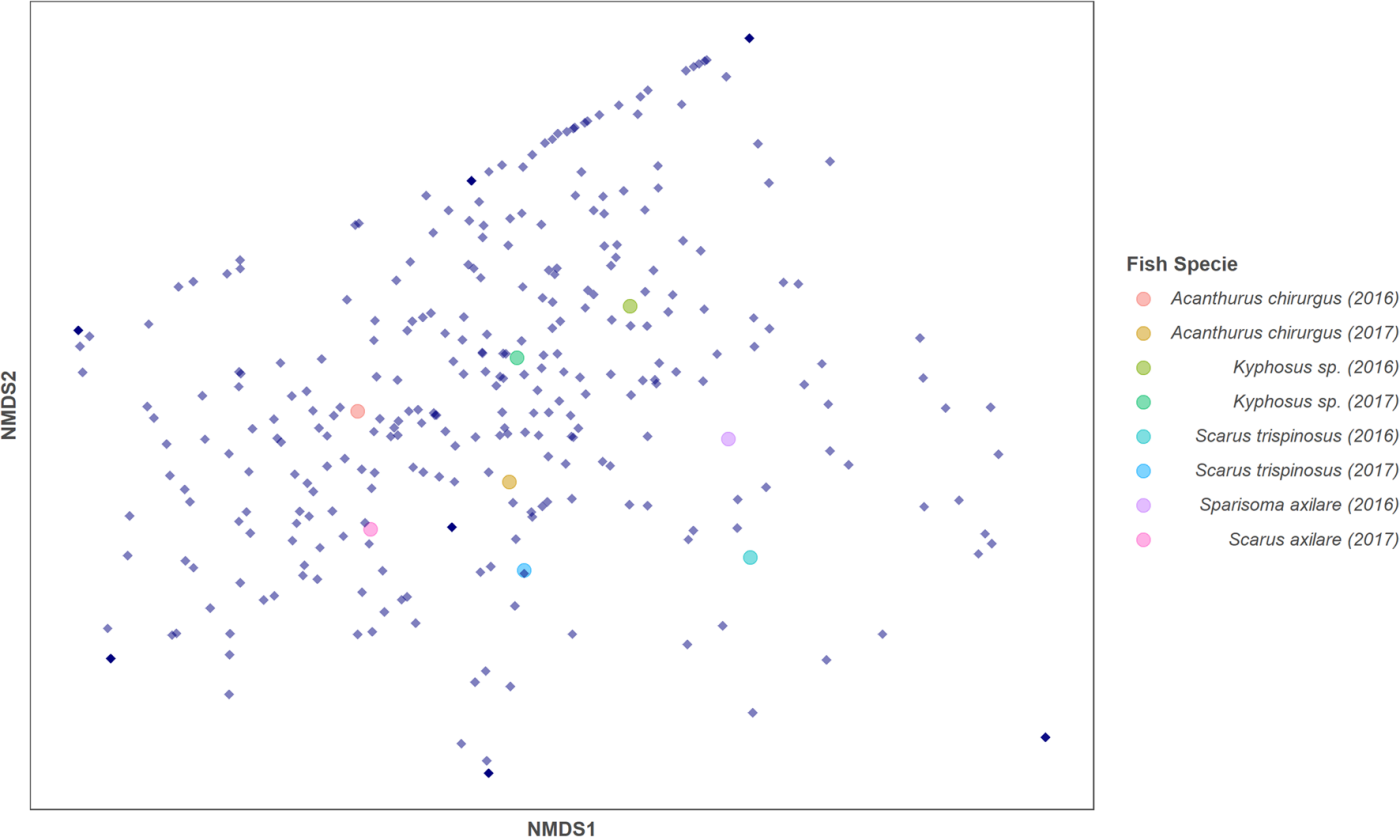
Non-metric Multidimensional Scaling (NMDS) with Bray–Curtis similarity index of gut content microbiomes. Approximately 440 family types contribute to form the dimensions of the graph with the Bray–Curtis index. The two specimens (gathered per year) of each fish, collected in 2016 and 2017, demonstrate that the *Acanthurus chirurgus* microbial group is closer to *Sparisoma axillare*, while the other two species have different patterns

**Fig. 6:**
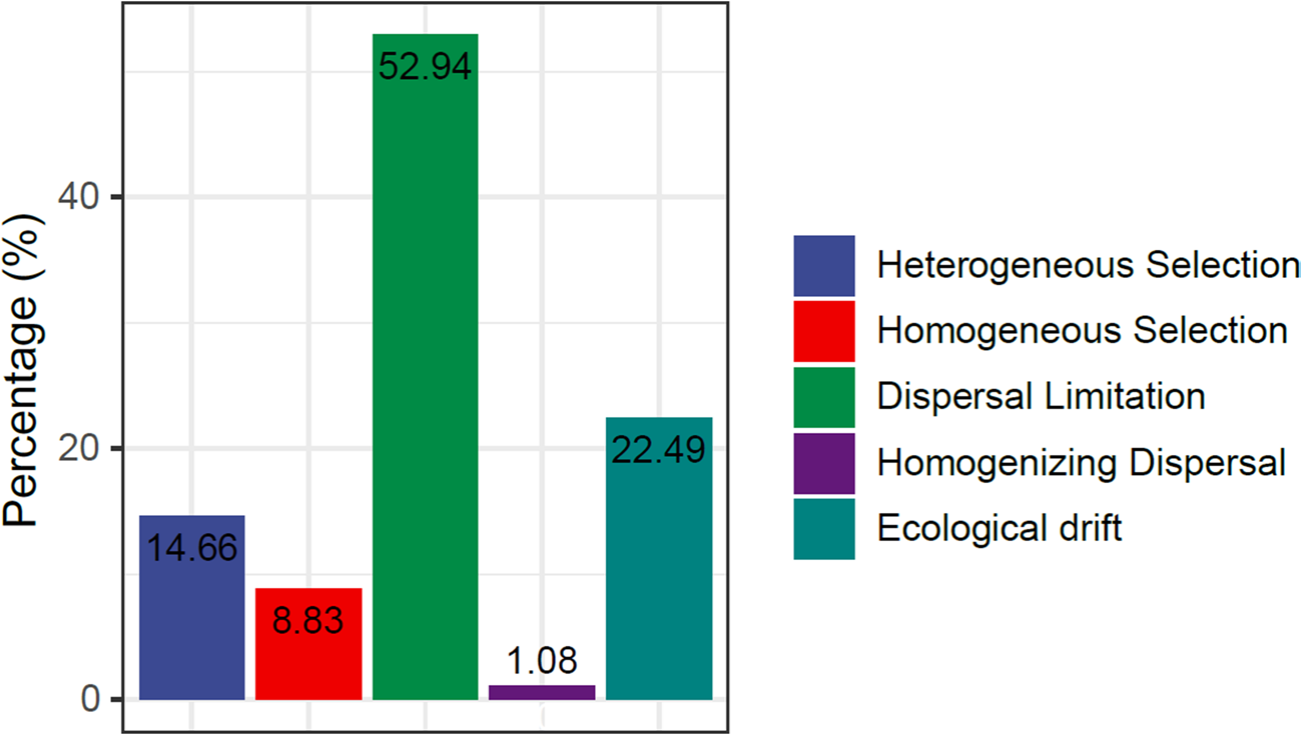
Evolutionary driving forces in fish microbiomes

**Fig. 7:**
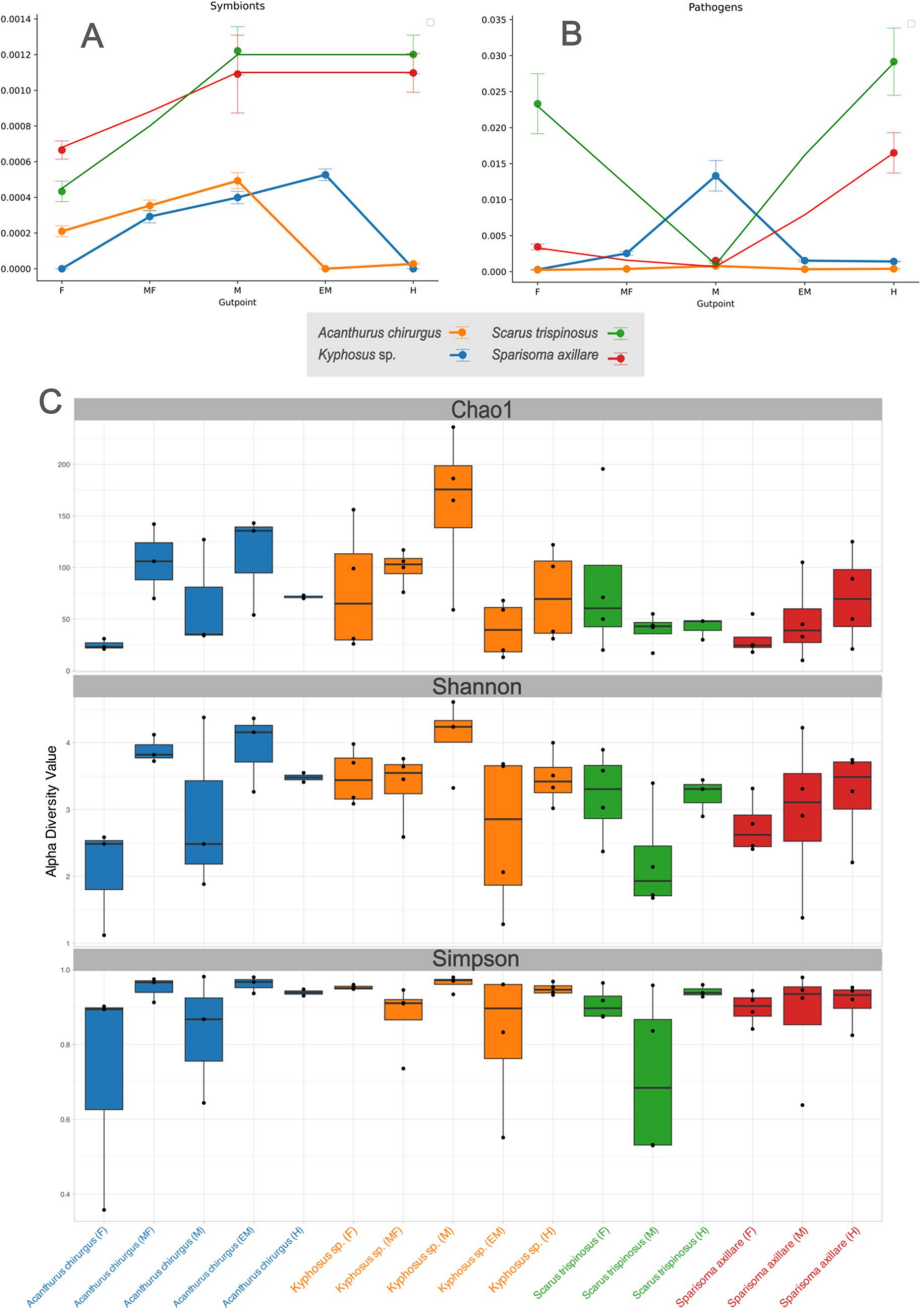
Abundance of potentially mutualistic (**A**) and pathogenic (**B**) bacteria for corals across the fish gut. Box plot of alpha diversity metrics (Chao1, Shannon, and Simpson) (**C**) for *Acanthurus chirurgus*, *Kyphosus* sp., *Scarus trispinosus*, and *Sparisoma axillare* at the prokaryotic genus level. F, foregut; MF, mid foregut; M, midgut; EM end midgut; H, hindgut. *Kyphosus* sp.: F = foregut/stomach, MF = anterior intestine, M = midgut, EM = end midgut, H = hindgut. *A. chirurgus*: F = foregut/anterior stomach, MF = gizzard-like stomach, M = anterior intestine, EM = midgut, H = hindgut. Coral mutualistic bacteria (*Ruegeria*, *Endozoycomonas*, and *Rhyzobia*). Coral pathogenic bacteria (*Vibrio*, *Photobacterium*, *Alteromonas*)

The midgut section of Kyphosus sp. showed the highest richness and diversity (Chao1 mean = 161.550 and Shannon mean = 4.101), followed by the midgut (Chao1 mean = 110.833 and Shannon = 3.928) and the gizzard-like stomach (Chao1 mean = 106.000 and Shannon = 3.888) of A. chirurgus. Finally, in the Simpson index, which evaluates dominant taxa of the microbiome, the midgut of Kyphosus sp. is the most dominant (Simpson mean = 0.963), followed by the end midgut of A. chirurgus (Simpson mean = 0.961) and the foregut of Kyphosus sp. (Simpson mean = 0.952) (Fig. 7C). Despite observed fluctuations in the abundance of gut microbiome components across different fish specimens and years, it is evident that each fish species has a unique microbiome profile (Fig. 5). To further understand the relative significance of different influencing factors on microbial evolution, we used a null model based on βNTI and RC-Bray metrics to study the roles of both stochastic processes (such as dispersal limitation, homogenizing dispersal, and ecological drift) and deterministic processes (like homogeneous and heterogeneous selection) [75]. The findings indicate that these forces are primarily driven by stochastic processes (Fig. 6). Specifically, among the four fish species, dispersal limitation exerted the most influence (52.94%) on microbial evolution. This suggests that each fish species co-diversifies with specific microbiomes. The most prevalent microbiome components are potentially stable symbionts, which have co-diversified to offer reciprocal benefits to the host and the microbiome.

The gut microbiome of a host significantly influences the immune system by stimulating the development of gut cells/tissues, the gut circulatory system, and the immune system itself [2, 74]. Gut microbes can generate a broad range of secondary metabolites, such as hormone precursors like tryptophan/serotonin, vitamins like B12, SCFAs like butyric, lactic, and propionic acids, 4-indolecarbaldehyde and L-3-phenyllactic acid. All of these contribute significantly to the host’s metabolism. Certain bacterial groups, such as *Prevotella* and *Bifidobacterium*, may influence nutrient production through fermentation, absorption, and body weight gain [79]. The Proteobacteria *Vibrionaceae*, *Rhodobacteraceae*, and *Fusobacteria* are noted to be abundant in fish guts and are potentially associated with fermentative processes [21, 82, 83]. Clostridia might also play a role in the fermentative digestion process in species like *Kyphosus* sp., *Sc. trispinosus*, and *A. chirurgus *[16, 29, 67]. Rhodobacteria may help in the assimilation of bile and cholesterol [1, 69], while *Fusobacteria* produces butyrate [3], a short-chain fatty acid often associated with carbohydrate fermentation, including that found in coral mucus [78, 80]. Butyrate, common in the intestines of herbivorous and omnivorous fish [15, 17], may inhibit the growth of potential fish pathogens [59]. *Scarus trispinosus* is known to prey on corals, including *Montastraea cavernosa* and *Mussismilia* [34, 35]. Notably, these corals have an abundant source of mucus composed of sulfated glycoproteins, sugars like fucose, and mucin proteins.

This study demonstrates that *Sc. trispinosus* and *Sp. axillare* may have a beneficial effect on coral reef health by dispersing potential mutualistic bacteria across the reef (Fig. 7A). However, these fish might also spread potentially harmful bacteria (Fig. 7B). Both *Kyphosus* sp. and *A. chirurgus* showed a reduced potential for spreading bacteria due to the lower occurrence of either mutualists or pathogens in their hindgut (Fig. 7). Corallivore feces are likely to contain a high volume of mutualistic bacteria (such as *Endozoicomonas*, *Ruegeria*, and *Rhyzobia*) and a lower volume of potential coral pathogens (vibrios) in comparison to the feces of algae grazers and detritivorous fish [24, 40]. The presence of ammonia-oxidizing Pirellulaceae and a low volume of Vibrionaceae in the gut of *Kyphosus* sp. suggests a complex microbial community, potentially influenced by the species' diet and habitat. Although these bacterial groups have been previously found in Abrolhos corals [27, 39], it is important to note that *Kyphosus* species are essentially herbivorous, primarily feeding on different types of algae. This indicates that the intestinal microbiome of these fish may also reflect the environmental bacteria present in their surroundings.

Corallivore feces, therefore, might contribute to coral health by supplying mutualistic microbes. Indeed, fish feces have been identified as a hotspot for *Symbiodinium* [42]. In corals, the growth of Rhodobacteraceae, Verrucomicrobiaceae, Flavobacteriaceae, Vibrionaceae, Fusobacteriaceae, Campylobacteraceae, and Cohaesibacteraceae is triggered by fish feces [23]. Fish that prey on corals showed an increase in potentially beneficial bacteria (such as *Oceanospirillum* and *Ruegeria*) and a decrease in opportunistic bacteria (such as Flammeovirgaceae, Rhodobacteraceae, Rhodospiralles, *Glaciecola*) [24]. The microbiome of the facultative coral-feeding butterflyfish (*Chaetodon capistratus*) varies across Caribbean reefs [18]. In degraded reefs (with low coral cover), microbiomes were more heterogeneous, enriched with fermentative bacteria and vibrios, and had fewer potential coral mutualists like *Endozoicomonas* [18].

Analysis of the foregut, midgut, and hindgut facilitated the assessment of potential variations in the microbiome composition throughout the entire fish gut (Fig. 7). Three microbial groups were differentiated within the Abrolhos fish gut microbiomes: (i) stable microbes (e.g., *Alteromonas*, *Vibrio*, and *Photobacterium* in *Kyphosus*), (ii) microbes with increased presence (e.g., *Ruegeria*, *Endozoycomonas*, and *Rhyzobia* in *Scarus* and *Sparissoma*), and (iii) microbes with decreased presence (e.g., *Ruegeria*, *Endozoycomonas*, and *Rhyzobia* in *Kyphosus* sp.) (Fig. 7). The observed variation in certain microbiome members suggests that the fish gut could act as a fermenter, encouraging the proliferation of specific microbial types (such as *Rhodobacteraceae*) and/or eliminating other bacteria throughout the gut. To further investigate the influence of food sources on the fish microbiome, isotopic analysis was conducted.

### The Isotopic Signature of the Gut Contents Supports Similarities in Feeding Among the Four Herbivorous Reef Fish

We obtained 44 isotopic signatures in total, which include gut content (20), fish muscular gut tissue (20), and food sources – namely, one rhodolith, one turf, and two corals (Table 1). The food sources composition was (δ13C and δ15N, ‰): *Montastraea cavernosa* (− 32.9 and 1.4), *Mussismilia braziliensis* (− 25.1 and 1.2), turf (− 19.1 and 1.8), rhodolith (− 12.8 and 5.0). Rhodolith exhibited the highest δ^13^C and δ^15^N values, while the *Mo. cavernosa* and *Mu. braziliensis* corals recorded the lowest. The δ^13^C values of fish gut content lay between − 23.1 and − 12.3‰. Observably, the isotopic signatures of *Kyphosus* sp. and *Sp. axillare* were different. Likewise, *Sparisoma*’s δ^13^C values and total tissue carbon levels differed significantly from those of *Scarus* and *Acanthurus*. We noted a significant variation in the gut content δ^13^C of *A. chirurgus* and *Kyphosus* sp. (ANOVA, *F* = 4.54, *p*-value = 0.017). The δ^13^C range for fish tissue was − 20.9 to − 10.2‰, with *Kyphosus* sp. showing a substantial difference in its tissue content δ^13^C compared to other fish (ANOVA, *F* = 15.74, *p*-value < 0.001).

**Table 1:** Taxa, tissue, number of samples (*n*), mean ± standard deviation, and enrichment factors (Ɛ) of δ^13^C and δ^15^N of *Sparisoma axillare*, *Scarus trispinosus*, *Acanthurus chirurgus*, and *Kyphosus* sp. at Abrolhos Bank, Southwestern Atlantic. Significant interspecific variations within a given tissue and intraspecific variations among different tissues are denoted by uppercase and lowercase letters, respectively (*p*-value < 0.05). *p-values refer to the differences between gut content and tissue for each fish species (δ^13^C/δ^15^N)

	Tissue	*n*	δ^13^C (‰)	δ^15^N (‰)	Ɛ δ^13^C (‰)	Ɛ δ^15^N (‰)	*p*-value*
*Kyphosus* sp.	Gut content	8	-14.4 ± 0.9^Aa^	3.0 ± 0.8^Aa^			0.07/0.000
	Tissue	8	-12.4 ± 0.8^Ab^	5.9 ± 0.8^Ab^	1.8	2.9	
*Acanthurus chirurgus*	Gut content	3	-19.8 ± 3.2^Ba^	2.7 ± 0.3^Aa^			0.03/0.005
	Tissue	3	-18.5 ± 3.7^Ba^	4.3 ± 0.5^Bb^	1.0	1.6	
*Scarus trispinosus*	Gut content	5	-17.9 ± 4.1^Aa^	2.7 ± 0.5^Aa^			0.01/0.000
	Tissue	5	-16.5 ± 1.1^BCa^	4.0 ± 0.3^Bb^	1.1	1.3	
*Sparisoma axillare*	Gut content	4	-15.1 ± 1.4^Aa^	2.9 ± 0.7^Aa^			0.14/0.007
	Tissue	4	-14.4 ± 1.8^Ca^	4.1 ± 0.2^Bb^	0.5	1.2	

The gut content δ^15^N varied between 1.7 and 4.0‰ and showed no significant difference among the four herbivorous fish (ANOVA, *F* = 0.26, *p*-value = 0.856). On the other hand, the fish tissue content δ^15^N ranged from 3.8 to 6.9‰. *Kyphosus* sp. possessed significantly higher δ^15^N compared to the other three fish species (ANOVA, F = 14.27, *p*-value < 0.001) (Table 1). It can be noted that *Kyphosus’*s habits distances itself from the other fish. Previous studies demonstrate seasonal shifts in diets are relevant. For instance, in summer, fish species are more generalist in their feeding characteristics. [20]. Remarkably, *Kyphosus* sp. was the only species displaying a significant difference in δ^13^C between gut and tissue contents (ANOVA, *F* = 19.36, *p*-value = 0.001). Comparable δ^13^C values between gut and tissue contents were noted in *A. chirurgus* (ANOVA, *F* = 0.22, *p*-value = 0.665), *Sc. Trispinosus* (ANOVA, *F* = 0.57, *p*-value = 0.470), and *Sp. axillare* (ANOVA, *F* = 0.38, *p*-value = 0.558). There was a higher δ^15^N in fish tissue relative to gut content for all fish species (Table 1; *Kyphosus* sp. – ANOVA, *F* = 3.76, *p*-value < 0.001; *A. chirurgus* – ANOVA, *F* = 2.49, *p*-value = 0.011; *Sc. Trispinosus* – ANOVA, *F* = 1.99, *p*-value = 0.001; *Sp. axillare* – ANOVA, *F* = 1.98, *p*-value = 0.015). The tissue and gut contents varied significantly in terms of δ^13^C and δ^15^N for all four species (Table 1). The highest discriminant factors were found in *Kyphosus* sp. (1.8 and 2.9‰, respectively), whereas *Sp. axillare* exhibited the lowest (0.5 and 1.2‰, respectively).

**Fig. 8:**
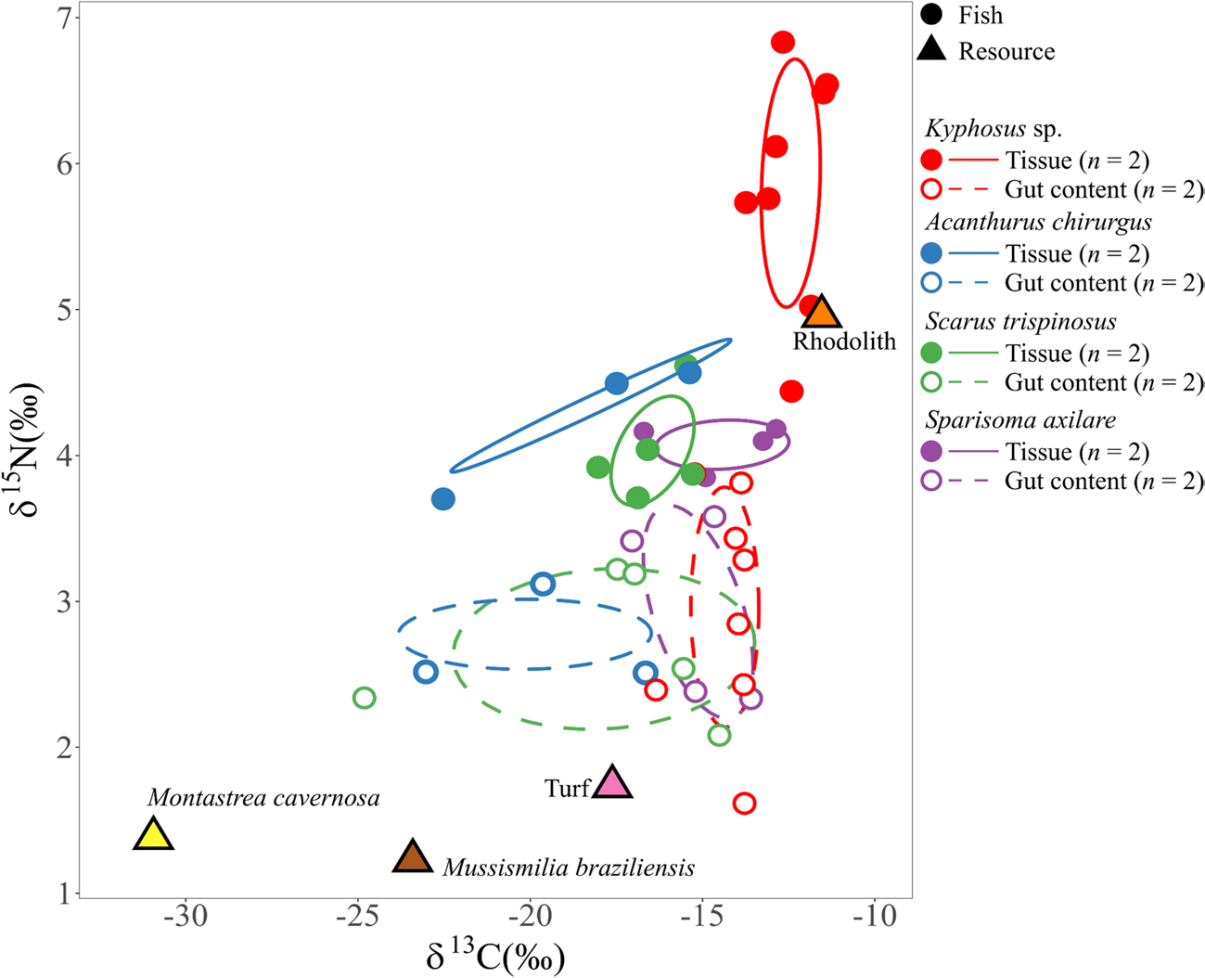
The standard ellipse area corrected for small samples (SEAc) of gut muscular tissue and gut content (microbiome fraction), both specified on Supplementary Table 2, for the four herbivorous fishes *Sparisoma axillare*, *Scarus trispinosus*, *Acanthurus chirurgus*, and *Kyphosus* sp. for each specimen (*n*) at Abrolhos Bank, Southwestern Atlantic

Some items in the diet of herbivorous fish, such as turf, brown algae, rodoliths, and corals, has been detailed (Fig. 8). As derived from the SIBER analysis, there was considerable overlap (> 64%) in gut contents between *Sc. Trispinosus* and *A. chirurgus*. This, along with similar CR, suggests that their food source is mainly turf (Fig. 8, Tables 2 and 3). Of note, *Sc. Trispinosus* demonstrated the most diverse and uneven diet among herbivorous fish, as evidenced by the highest values for CR, TA, SEAc, MNND, and SDNND in gut content (Table 3).

**Table 2:** Overlapping niche (SEAc, %) among reef fishes. Values are gut contents/tissue

	*Kyphosus* sp.	*A. chirurgus*	*S. trispinosus*	*S. axillare*
Gut contents				
*Kyphosus* sp.	-/-	-/-	45.9/-	60.4/-
*Acanthurus chirurgus*	-/-	-/-	79.5/-	-/-
*Scarus trispinosus*	13.6/-	64.5/-	-/-	27.3/22.4
*Sparisoma axillare*	40.4/-	-/-	61.4/25.9	-/-

**Table 3:** Isotopic niche metrics. Significant interspecific variations within a given tissue and intraspecific variations among different tissues are denoted by uppercase and lowercase letters, respectively (*p*-value < 0.05). Abbreviations definitions (NR, CR, TA, SEAc, CD, MNND, and SDNND) in the text. Values are gut contents/gut muscle tissue

	NR (‰)	CR (‰)	TA (‰)	SEAc (‰^2^)	CD (‰)	MNND (‰)	SDNND (‰)
*Kyphosus* sp.	2.2/2.3	2.4/2.2	2.8/3.0	2.5/2.3	1.0^Aa^/1.0^Aa^	0.5^Aa^/0.5^Aa^	0.4^Aa^/0.2^Ba^
*Acanthurus chirurgus*	0.6/0.8	6.1/6.9	1.8/0.6	6.9/2.3	2.2^Aa^/2.6^Ba^	3.0^Ba^/3.0^Ba^	0.2^Aa^/1.6^Ab^
*Scarus trispinosus*	1.1/0.9	9.9/2.6	5.5/1.2	8.5/1.4	2.7^Aa^/0.8^Aa^	2.0^ABa^/0.7^Aa^	2.8^Ba^/0.2^Bb^
*Sparisoma axillare*	1.2/0.3	3.3/3.7	2.2/0.6	3.7/1.2	1.2^Aa^/1.3^ABa^	1.5^ABa^/1.0^Aa^	0.3^Aa^/0.7^Aa^

In contrast, *Kyphosus* sp. and *Sp. axillare* showed isotopic niche overlaps between 40 to 60%, indicating mutual reliance on a food source associated to turf and rodolith, backed by high NR values. As uncovered from the fish tissue’s isotopic profiles, *A. chirurgus* and *Kyphosus* sp. displayed distinct ellipses in their isotopic niches, indicating no overlap with other species. This implies unique assimilation patterns. On the other hand, *Sp. axillare* and *Sc. Trispinosus* exhibited overlap between 22 and 26%. Traces of *M. braziliensis* coral, turf, and rhodoliths consumption were found in *A. chirurgus*. In contrast, *Kyphosus* sp. appeared to favor resources associated to rodolith, demonstrated by the lower CD values and the lowest MNND and SDNND values.

A recent study established that the isotopic signatures of *A. chirurgus* (δ^13^C: − 18.7 ± 0.3‰; δ^15^N: 12.3 ± 0.2‰) and *Sp. axillare* (δ^13^C: − 16.0 ± 0.1‰; δ^15^N: 10.8 ± 0.2‰) varied in an upwelling subtropical region [9]. However, this study found both fish species to have similar nitrogen isotopic signatures,*A. chirurgus* had a mean ± standard deviation (stdev) of δ^13^C − 18.5 ± 3.7‰ and δ^15^N 4.3 ± 0.5‰, while *S. axillare* showed δ^13^C − 14.4 ± 1.8‰ and δ^15^N 4.1 ± 0.2‰. The decreased δ^15^N in *A. chirurgus* tissue from Abrolhos tropical reefs compared to the upwelling region could potentially be due to higher nitrogen levels in upwelling food items. The gut content’s δ^13^C and δ^15^N signatures were similar among the four species in Abrolhos. Even though these herbivorous fish consume some similar food sources in Abrolhos, differences in their microbiomes, host genetic backgrounds, and metabolic rates may contribute to the diversity in nutrient assimilation, resulting in varying tissue isotopic signatures.

## Conclusions

This study highlights key features of the herbivorous fish gut microbiome in Abrolhos. Firstly, the study reveals the potential role of reef fish, specifically *Scarus trispinosus* and *Sparisoma axillare* parrotfish, in spreading mutually beneficial and potentially harmful bacteria. However, due to the drastic reduction in these fish populations as a result of overfishing, their role in disseminating these bacteria on the Abrolhos reefs is likely diminished. Given the rising local and global changes threatening the Abrolhos reefs, the management of fisheries must be reviewed to mitigate their negative impact. Secondly, the study identifies a partial overlap in isotopic niches among four fish species, suggesting similarities in feeding. This similarity also implies that microbes might play a role in isotopic partitioning within the fish tissues. Though these fish feed from the same resource pool, each species may depend on its unique microbiome for isotopic differentiation. This suggests that gut microbiomes could be driving forces in differentiating fish tissue isotopic signatures. Bacteria associated with fermentation and nutrient absorption within the microbiome could play pivotal roles in host health and metabolism. This study underscores the importance of considering not only the fish feeding but also the influence of their microbiomes on reef ecology and health. These findings pave the way for future research and more effective conservation strategies.

### Supplementary Information

Below is the link to the electronic supplementary material.

Supplementary file1 (DOCX 2776 KB)

## Data Availability

Data is provided within the manuscript or supplementary information files. Extra data available upon request. Raw data have been deposited in GenBank, https://ncbi.nlm.nih.gov, under BioProject accession number PRJNA1131363: Biosample SAMN42278458 to SAMN42278510.
