# Supplementary material for: The Abrolhos Nominally Herbivorous Coral Reef Fish Acanthurus chirurgus, Kyphosus sp., Scarus trispinosus, and Sparisoma axillare Have Similarities in Feeding But Species-Specific Microbiomes
Source: Microb Ecol. 2024 Aug 31;87(1):110. doi: 10.1007/s00248-024-02423-x (PMC11365853; doi:10.1007/s00248-024-02423-x)
Supplement: Supplementary file 1 — Supplementary file1 (DOCX 2776 KB) [file 248_2024_2423_MOESM1_ESM.docx]

**Supplementary table 1.** List of 16S rRNA libraries obtained in this study.

| **Fish species** | **Gut portion** | **Sampling year** | **# Reads** | **#OTUs** |
| --- | --- | --- | --- | --- |
| *Acanthurus chirurgus* | Foregut = Anterior stomach (F) | 2017 | 226810 | 5534 |
| *Acanthurus chirurgus* | Foregut = Anterior stomach (F) | 2016 | 118140 | 7291 |
| *Acanthurus chirurgus* | Foregut = Anterior stomach (F) | 2017 | 118584 | 4398 |
| *Acanthurus chirurgus* | Gizzard-like stomach (MF) | 2017 | 243876 | 12242 |
| *Acanthurus chirurgus* | Gizzard-like stomach (MF) | 2017 | 221082 | 21058 |
| *Acanthurus chirurgus* | Gizzard-like stomach (MF) | 2016 | 126452 | 6237 |
| *Acanthurus chirurgus* | Anterior intestine (M) | 2016 | 289016 | 7668 |
| *Acanthurus chirurgus* | Anterior intestine (M) | 2017 | 125996 | 9291 |
| *Acanthurus chirurgus* | Anterior intestine (M) | 2016 | 120604 | 11360 |
| *Acanthurus chirurgus* | Midgut (EM) | 2017 | 287338 | 14165 |
| *Acanthurus chirurgus* | Midgut (EM) | 2016 | 140614 | 11991 |
| *Acanthurus chirurgus* | Midgut (EM) | 2017 | 125712 | 13490 |
| *Acanthurus chirurgus* | Hindgut (H) | 2017 | 115874 | 9829 |
| *Acanthurus chirurgus* | Hindgut (H) | 2017 | 123150 | 9126 |
| *Kyphosus* sp. | Foregut = Stomach (F) | 2017 | 121586 | 3602 |
| *Kyphosus* sp. | Foregut = Stomach (F) | 2016 | 119372 | 13189 |
| *Kyphosus* sp. | Foregut = Stomach (F) | 2016 | 59091 | 3011 |
| *Kyphosus* sp. | Foregut = Stomach (F) | 2016 | 118140 | 33871 |
| *Kyphosus* sp. | Anterior intestine (MF) | 2017 | 229964 | 11882 |
| *Kyphosus* sp. | Anterior intestine (MF) | 2016 | 140614 | 15555 |
| *Kyphosus* sp. | Anterior intestine (MF) | 2017 | 83466 | 16330 |
| *Kyphosus* sp. | Midgut (M) | 2017 | 83466 | 7183 |
| *Kyphosus* sp. | Midgut (M) | 2017 | 173197 | 37578 |
| *Kyphosus* sp. | Midgut (M) | 2017 | 90836 | 29088 |
| *Kyphosus* sp. | End-Midgut (EM) | 2017 | 123150 | 8041 |
| *Kyphosus* sp. | End-Midgut (EM) | 2016 | 289016 | 8327 |
| *Kyphosus* sp. | End-Midgut (EM) | 2017 | 71378 | 8277 |
| *Kyphosus* sp. | End-Midgut (EM) | 2016 | 70323 | 759 |
| *Kyphosus* sp. | Hindgut (H) | 2017 | 66711 | 3549 |
| *Kyphosus* sp. | Hindgut (H) | 2016 | 88287 | 13301 |
| *Scarus trispinosus* | Foregut (F) | 2016 | 877114 | 3929 |
| *Scarus trispinosus* | Foregut (F) | 2017 | 114704 | 34112 |
| *Scarus trispinosus* | Foregut (F) | 2017 | 223946 | 8817 |
| *Scarus trispinosus* | Foregut (F) | 2016 | 1441505 | 1697 |
| *Scarus trispinosus* | Midgut (M) | 2017 | 115874 | 5009 |
| *Scarus trispinosus* | Midgut (M) | 2016 | 1227761 | 5362 |
| *Scarus trispinosus* | Midgut (M) | 2017 | 173539 | 16166 |
| *Scarus trispinosus* | Midgut (M) | 2016 | 1476372 | 3918 |
| *Scarus trispinosus* | Hindgut (H) | 2016 | 1260090 | 3312 |
| *Scarus trispinosus* | Hindgut (H) | 2017 | 33390 | 11352 |
| *Scarus trispinosus* | Hindgut (H) | 2016 | 748802 | 1529 |
| *Sparisoma axillare* | Foregut (F) | 2017 | 71378 | 6248 |
| *Sparisoma axillare* | Foregut (F) | 2016 | 837515 | 1463 |
| *Sparisoma axillare* | Foregut (F) | 2017 | 67155 | 10931 |
| *Sparisoma axillare* | Foregut (F) | 2016 | 849835 | 1707 |
| *Sparisoma axillare* | Midgut (M) | 2017 | 49956 | 8476 |
| *Sparisoma axillare* | Midgut (M) | 2016 | 1607531 | 1830 |
| *Sparisoma axillare* | Midgut (M) | 2017 | 83466 | 6087 |
| *Sparisoma axillare* | Midgut (M) | 2016 | 183850 | 4465 |
| *Sparisoma axillare* | Hindgut (H) | 2017 | 115874 | 18488 |
| *Sparisoma axillare* | Hindgut (H) | 2016 | 808783 | 6946 |
| *Sparisoma axillare* | Hindgut (H) | 2017 | 49956 | 5439 |
| *Sparisoma axillare* | Hindgut (H) | 2016 | 73272 | 4234 |

**Supplementary table 2.** List of individual δ^13^C and δ^15^N by matrix, gut portion and sampling year in this study.

| **Fish species** | **Specimen** | **Code** | **Matrix** | **Gut portion** | **Sampling year** | **δ^13^C** | **δ^15^N (‰)** |
| --- | --- | --- | --- | --- | --- | --- | --- |
| *Acanthurus chirurgus* | #1 | AAMP | Gut content | End-Midgut |  | -16,7 | 2,5 |
| *Acanthurus chirurgus* | #2 | AAMP | Gut content | End-Midgut |  | -19,6 | 3,1 |
| *Acanthurus chirurgus* | #2 | AAP | Gut content | Hindgut |  | -23,0 | 2,5 |
| *Acanthurus chirurgus* | #1 | AAMP | Tissue | End-Midgut |  | -15,4 | 4,6 |
| *Acanthurus chirurgus* | #2 | AAMP | Tissue | End-Midgut |  | -22,5 | 3,7 |
| *Acanthurus chirurgus* | #2 | AAP | Tissue | Hindgut |  | -17,5 | 4,5 |
| *Kyphosus* | #1 | KAA | Gut content | Foregut |  | -16,3 | 2,4 |
| *Kyphosus* | #1 | KAMA | Gut content | Mid-Foregut |  | -13,9 | 3,8 |
| *Kyphosus* | #1 | KAMM | Gut content | Median-Midgut |  | -14,0 | 2,8 |
| *Kyphosus* | #1 | KAMP | Gut content | End-Midgut |  | -14,0 | 3,4 |
| *Kyphosus* | #1 | KAP | Gut content | Hindgut |  | -13,8 | 2,4 |
| *Kyphosus* | #2 | KAMM | Gut content | Median-Midgut |  | -15,2 | 3,9 |
| *Kyphosus* | #2 | KAMP | Gut content | End-Midgut |  | -13,8 | 1,6 |
| *Kyphosus* | #2 | KAP | Gut content | Hindgut |  | -13,8 | 3,3 |
| *Kyphosus* | #1 | KAA | Tissue | Foregut |  | -12,7 | 6,8 |
| *Kyphosus* | #1 | KAMA | Tissue | Mid-Foregut |  | -13,1 | 5,8 |
| *Kyphosus* | #1 | KAMM | Tissue | Median-Midgut |  | -11,4 | 6,5 |
| *Kyphosus* | #1 | KAMP | Tissue | End-Midgut |  | -13,7 | 5,7 |
| *Kyphosus* | #1 | KAP | Tissue | Hindgut |  | -12,9 | 6,1 |
| *Kyphosus* | #2 | KAMM | Tissue | Median-Midgut |  | -12,4 | 4,4 |
| *Kyphosus* | #2 | KAMP | Tissue | End-Midgut |  | -11,9 | 5,0 |
| *Kyphosus* | #2 | KAP | Tissue | Hindgut |  | -11,5 | 6,5 |
| *Scarus trispinosus* | #1 | SCAA | Gut content | Foregut |  | -14,5 | 2,1 |
| *Scarus trispinosus* | #1 | SCAM | Gut content | Midgut |  | -24,8 | 2,3 |
| *Scarus trispinosus* | #1 | SCAP | Gut content | Hindgut |  | -17,5 | 3,2 |
| *Scarus trispinosus* | #2 | SCAA | Gut content | Foregut |  | -15,6 | 2,5 |
| *Scarus trispinosus* | #2 | SCAM | Gut content | Midgut |  | -17,0 | 3,2 |
| *Scarus trispinosus* | #1 | SCAA | Tissue | Foregut |  | -16,6 | 4,0 |
| *Scarus trispinosus* | #1 | SCAM | Tissue | Midgut |  | -15,3 | 3,9 |
| *Scarus trispinosus* | #1 | SCAP | Tissue | Hindgut |  | -18,0 | 3,9 |
| *Scarus trispinosus* | #2 | SCAA | Tissue | Foregut |  | -16,9 | 3,7 |
| *Scarus trispinosus* | #2 | SCAM | Tissue | Midgut |  | -15,5 | 4,6 |
| *Sparisoma axillare* | #1 | SPAA | Gut content | Foregut |  | -15,2 | 2,4 |
| *Sparisoma axillare* | #1 | SPAM | Gut content | Midgut |  | -14,7 | 3,6 |
| *Sparisoma axillare* | #2 | SPAA | Gut content | Foregut |  | -13,6 | 2,3 |
| *Sparisoma axillare* | #2 | SPAM | Gut content | Midgut |  | -17,0 | 3,4 |
| *Sparisoma axillare* | #1 | SPAA | Tissue | Foregut |  | -16,7 | 4,2 |
| *Sparisoma axillare* | #1 | SPAM | Tissue | Midgut |  | -13,2 | 4,1 |
| *Sparisoma axillare* | #2 | SPAA | Tissue | Foregut |  | -14,9 | 3,9 |
| *Sparisoma axillare* | #2 | SPAM | Tissue | Midgut |  | -12,9 | 4,2 |
